# Investigating the Potential for Ultraviolet Light to Modulate Morbidity and Mortality From COVID-19: A Narrative Review and Update

**DOI:** 10.3389/fcvm.2020.616527

**Published:** 2020-12-23

**Authors:** Shelley Gorman, Richard B. Weller

**Affiliations:** ^1^Telethon Kids Institute, University of Western Australia, Perth, WA, Australia; ^2^Centre for Inflammation Research, University of Edinburgh, Edinburgh, United Kingdom

**Keywords:** COVID-19, SARS-CoV-2, ultraviolet light, sun exposure, vitamin D, nitric oxide

## Abstract

During the COVID-19 (coronavirus disease of 2019) pandemic, researchers have been seeking low-cost and accessible means of providing protection from its harms, particularly for at-risk individuals such as those with cardiovascular disease, diabetes and obesity. One possible way is via safe sun exposure, and/or dietary supplementation with induced beneficial mediators (e.g., vitamin D). In this narrative review, we provide rationale and updated evidence on the potential benefits and harms of sun exposure and ultraviolet (UV) light that may impact COVID-19. We review recent studies that provide new evidence for any benefits (or otherwise) of UV light, sun exposure, and the induced mediators, vitamin D and nitric oxide, and their potential to modulate morbidity and mortality induced by infection with SARS-CoV-2 (severe acute respiratory disease coronavirus-2). We identified substantial interest in this research area, with many commentaries and reviews already published; however, most of these have focused on vitamin D, with less consideration of UV light (or sun exposure) or other mediators such as nitric oxide. Data collected to-date suggest that ambient levels of both UVA and UVB may be beneficial for reducing severity or mortality due to COVID-19, with some inconsistent findings. Currently unresolved are the nature of the associations between blood 25-hydroxyvitamin D and COVID-19 measures, with more prospective data needed that better consider lifestyle factors, such as physical activity and personal sun exposure levels. Another short-coming has been a lack of measurement of sun exposure, and its potential to influence COVID-19 outcomes. We also discuss possible mechanisms by which sun exposure, UV light and induced mediators could affect COVID-19 morbidity and mortality, by focusing on likely effects on viral pathogenesis, immunity and inflammation, and potential cardiometabolic protective mechanisms. Finally, we explore potential issues including the impacts of exposure to high dose UV radiation on COVID-19 and vaccination, and effective and safe doses for vitamin D supplementation.

## Introduction

The first cases of COVID-19 (coronavirus disease of 2019) presented in Wuhan, China in December 2019. Since then, COVID-19 has become a global pandemic, occurring on every continent except Antarctica with >54 million confirmed cases and >1.3 million deaths (18th November 2020, https://covid19.who.int/) worldwide. Few treatments other than supportive care are (currently) available, although there are likely benefits for repurposed drugs, such corticosteroids (which have anti-inflammatory effects) for reducing all-cause mortality in critically ill COVID-19 patients (1), and multiple promising vaccine candidates. The progression of COVID-19 is characterized by three phases. First, angiotensin-converting enzyme 2 (ACE2)+ nasal epithelial cells (pre/asymptomatic phase) are infected with SARS-CoV-2 (severe acute respiratory disease coronavirus-2). Then, the infection spreads to ACE2+ type II alveolar epithelial cells (pneumonitis). Finally, disruption of the epithelial-endothelial barrier occurs with complement deposition and hyperinflammation (severe COVID-19) (2). People at-risk of developing severe COVID-19 and fatal outcomes include patients with cardiovascular disease, diabetes, hypertension and/or obesity (3–5). This increased risk may be related to the low-grade inflammation that characterizes these chronic diseases, age-related reductions in anti-viral immunity, expression of ACE2 in vulnerable tissues (e.g., adipose, heart), underlying tissue fibrosis, impairments in lung function, and non-medical factors (e.g., poverty, crowding). For more information on other aspects of SARS-CoV-2 and the COVID-19 pandemic, including aspects of viral epidemiology and evolution, disease pathogenesis, prevention and treatment, please refer to comprehensive reviews (6, 7).

There is emerging evidence that sun exposure and UV light may have beneficial effects in preventing cardiometabolic dysfunction (8, 9). While there has been significant commentary on the potential benefits of the UV-induced mediator vitamin D (also reviewed below), the direct effects of exposure to UV light on COVID-19 has received less attention. Historical use of phototherapy and sun exposure to treat tuberculosis (10) suggests that there are likely benefits. Here, we provide an update of newly acquired knowledge (as of 18th November 2020), first describing beneficial associations between lower latitudes and increased ambient UV levels and COVID-19-related outcomes, and the capacity for germicidal UVC (254 nm) radiation to inactivate the SARS-CoV-2 virus. We also consider the potential harms of excessive sun exposure for both COVID-19 disease and vaccination efficacy, contrasted with possible benefits of low level (non-burning) sun exposure for those with cardiometabolic dysfunction. Finally, we review new knowledge around the potential for “beneficial mediators” induced by exposure to UV light on COVID-19, specifically vitamin D and nitric oxide. We describe possible anti-viral, anti-inflammatory and cardiometabolic beneficial means through which controlled exposure to UV light, or interventions that administer these beneficial mediators could be harnessed to combat COVID-19. To provide this update, a literature search was conducted on PubMed (until 18th November 2020) in which the following keywords were combined: (COVID-19 OR social distancing) AND (season, latitude, ultraviolet, sun exposure, sunlight, solar, phototherapy, vitamin D OR nitric oxide). While we focus on UV light and sun exposure, there may also be effects of other wavelengths within the solar spectrum, including violet/blue (400–700 nm), red (600–700 nm), and infrared (700–1,000 nm) light on COVID-19 (11) as well as preventative and therapeutic possibilities for other light-based therapies (12).

## UV Light, Sun Exposure, and COVID-19

**Figure 1:**
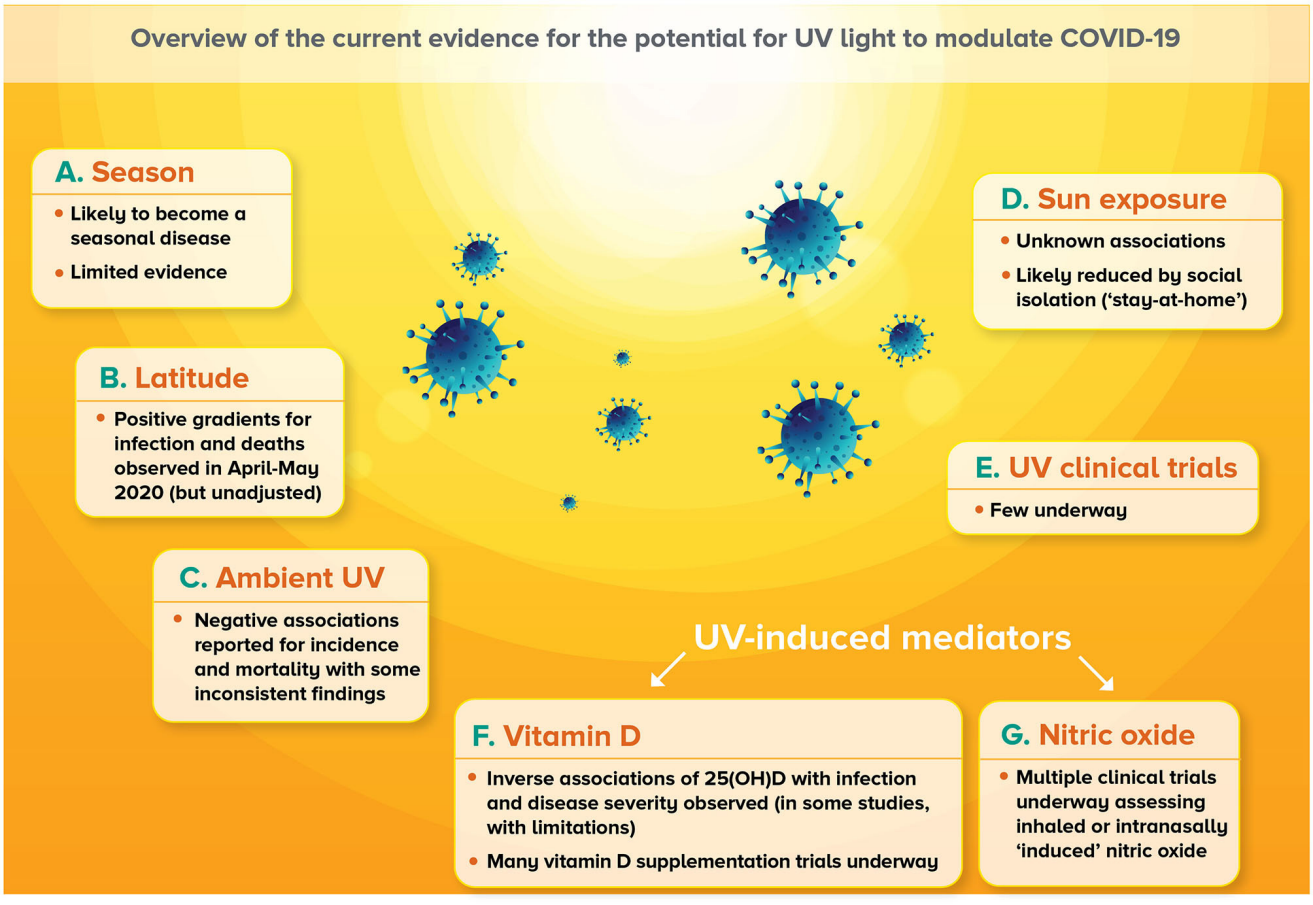
Overview of current evidence for the potential for UV light to modulate COVID-19. **(A)** Infections with SARS-CoV-2 are predicted to establish seasonality, although there is limited evidence for this as yet. **(B)** Positive latitude gradients for COVID-19-related outcomes were observed in April–May 2020, although these findings were largely unadjusted for country-wide differences in public health measures as well as other important factors. **(C)** Negative associations between ambient UVA or UVB levels and COVID-19-related outcomes have been observed with some inconsistent findings, particularly for incidence. **(D)** Little information has been published around the associations between sun exposure and COVID-19, although it is likely that social isolation (“stay-at-home”) orders implemented by many legislatures limited opportunities for sun exposure. **(E)** Very few clinical trials are currently underway investigating whether deliberate exposure to UV radiation, or sunlight could affect COVID-19 outcomes. **(F)** Some emerging evidence suggests inverse correlations between circulating 25-hydroxyvitamin D [25(OH)D] levels with COVID-19 outcomes, although findings are limited in their scope. There are also many clinical trials that are testing the capacity of supplementation with vitamin D to reduce the incidence and severity of COVID-19. **(G)** A number of clinical trials are also underway assessing the potential for inhaled nitric oxide or nitric oxide intra-nasally generated or released (“induced”) by application of a chemical.

Sunlight is composed of a spectrum of light, of which the UV component can be divided into 3 bandwidths: UVA (315–400 nm), UVB (280–315 nm), and UVC (100–280 nm) radiation. All UVC and most UVB (~90%) does not reach the Earth's surface as these wavelengths of light are blocked by oxygen in the atmosphere. Most of the UV light that does reach the surface of the Earth (terrestrial UV) is UVA (~95%), and the rest is UVB. Harmful effects of excessive exposure to UV light, including sunburn, skin cancers and eye disease are well-known, and the reader is directed toward recent comprehensive reviews of the impacts of UV radiation and sun exposure on human health and disease, including those that consider infectious disease, viral infection and vaccination (13, 14). In this section, we review new findings describing the nature of the associations between proxies for sun exposure (including: season, latitude, ambient UV levels) and COVID-19, describe any clinical trials underway testing the impacts of UV light, and review findings related to the effects of social distancing measures on sun exposure levels. For a “higher-level” overview of these new findings, please see Figure 1.

### Season and SARS-CoV-2

Seasonal variations in disease incidence can be caused by environmental, behavioral, and immunological factors with the relative importance of these varying by location and disorder (15). Many infectious diseases show seasonal variations (16) in incidence and mortality, including those caused by coronaviruses (17, 18). Seasonality for coronaviruses may be more likely in temperate climates (which have more distinct seasons and wider temperature ranges) than tropical climates (19). This seasonality can be explained by variations in the effective reproductive number and population level susceptibility (20), which may be influenced by the length of protective immunity induced by infection (17). Natural oscillations in temperature (21), humidity (22, 23), and UV radiation (24) correlate with variations in incidence of several infectious diseases. Observational studies show that seasonal influenza incidence is affected by solar radiation, humidity and temperature (22–24). During the 1918–1919 influenza pandemic, estimated UVB doses in US summer (July) correlated with reduced case fatality and pneumonia rates (25). However, insufficient time has passed to determine whether SARS-CoV-2 infections and COVID-19 have seasonal variation, and whether increased ambient UV levels in summer are protective (26), although some modeling studies predict that SARS-CoV-2 infections will fall into seasonal patterns with wintertime outbreaks (27) in 2020 in the northern hemisphere (28).

### Latitude and COVID-19

Terrestrial UV levels reduce with increasing distance from the equator (i.e., latitude). A positive gradient between mortality rates for COVID-19 and latitude was reported in mid-April 2020, with increased deaths observed for (mainly) European countries at latitudes >35°N (29). Similar observations were also made in the USA around this time (30). By April 2 2020, using country-based data from the World Health Organization, positive associations between latitude and COVID-19 cases (*r* = 0.54, *p* < 0.01) or deaths (*r* = 0.38, *p* < 0.01) were reported (31). Similar findings were observed for data collected from 25 areas in the USA and Europe until April 30 2020 (32), and for 88 countries on May 17 2020 (33). Caution should be used when interpreting these findings, as they may be indicative of the progression of the pandemic from northern to southern countries across this time period. Furthermore, some of these studies were limited by the lack of consideration of country-specific differences in public health measures undertaken to limit viral spread, such as social distancing strategies and infection testing rates, as well as other important factors such as: socioeconomic status, population density, urban connectivity, age, gender and comorbidities (34). Indeed, when similar confounders were included in a multivariable analysis, no significant association for latitude and COVID-19 case rates was observed in a worldwide study of 144 geopolitical areas in March 2020 (35).

### Ambient UV Levels, and COVID-19 Incidence and Deaths

“Beneficial” inverse associations have been observed between higher ambient UV levels and lower COVID-19 incidence in most, but not all studies. Incident UV light levels correlated inversely with the peak rate of rise in SARS-CoV-2 infections, with temperature and humidity having smaller effects, using data collected from 128 countries and 98 states/provinces until April 2020 (28). Similarly, in a study of 359 regions from China, Italy, USA, Spain, Canada, and Australia, average solar irradiance (W/m^2^) and UV Index (erythemally weighted for UVB exposure) inversely correlated with COVID-19 cases per 100,000 individuals on March 23rd 2020 (36). Using data from 173 countries, across 3,235 regions, surface UV intensity levels were estimated to reduce the daily growth rate in COVID-19 cases with this effect estimation likely weakened by location-specific social distancing strategies (37). A higher UV Index also associated with lower rates of new cases in a state-based study in the USA, independent of gross domestic product (GDP), obesity rates and age-related factors in January–March 2020 (38). Significant associations between higher UV Index and lower COVID-19 prevalence were observed in Japanese prefectures (39), and 33 cities in the USA (40). Some negative associations were also reported for reduced infection rates and increased ambient UV levels measured in 5 Brazilian cities (March–July 2020) (41) and selected counties of north-east and central-mid-west (census regions 1 and 2) of the USA (April–July 2020) (42).

However, no significant associations were observed between 7 or 14 day-lagged measurements of the UV Index and COVID-19 incidence, following adjustment for other environmental factors as well as GDP and global health security index for data collected across 206 countries/regions (January–April 2020) (43). Similarly, in a study of people living in 224 Chinese cities, no significant associations between ambient UV levels and COVID-19 incidence rate (January–March 2020) were observed (44). There was also no significant association between solar radiation and COVID-19 incidence across 31 regions in Iran (February–March 2020) (45). Finally, levels of solar radiation or sunshine hours positively associated with COVID-19 infections or incidence in India (until the 27th April 2020) (46) and Spain (March–April 2020) (47), respectively.

Some “beneficial” inverse associations of higher ambient UV levels and lower COVID-19 mortality have been detected in most studies done to-date. We observed a dose-dependent correlation between average ambient UVA levels (from satellite-derived data, measured in January-April 2020) and COVID-19 deaths, in a trio of studies carried out in the USA, United Kingdom and Italy, then pooled to show a reduced mortality risk ratio of 0.68 (95% CI 0.53–0.89) per 100 kJ/m^2^ increase in mean daily UV (48) (preprint). These data were corrected for the vitamin D-weighted solar spectrum (excluding the vitamin D-forming UVB component). A zero-inflated negative binomial model was used which corrects firstly for the likelihood of encountering and being infected with SARS-CoV-2 (considering population density, transport patterns and prevalence of COVID-19 within that population) and then for factors known to confound outcomes of COVID-19 (including age, ethnicity, socio-economic status, pollution). These data suggest that there are benefits from UV light independently of vitamin D formation in reducing COVID-19 mortality. Benefits for the UVB component of sunlight have also been reported, in which a negative association between the UV Index and COVID-19 deaths were observed in data from 152 countries using modeling that considered other potential effects of local weather (e.g., temperature, humidity) across January–May 2020 (49). Beneficial inverse associations between COVID-19 severity or death and local ambient UV levels or annual mean sunlight hours were also observed in Spain (50) and France (51), respectively, although this French study has been critiqued for the statistical approach undertaken (52). However, GDP and body mass index, but not mean UV Index (measured across November 2019–April 2020) were significant predictors of COVID-19 deaths (per million) in a multivariate regression analysis across the world (53).

There is uncertainty of the nature of the associations between ambient UV levels and the basic reproduction number (R_0_) of SARS-CoV-2. Significant inverse correlations between UV Index and SARS-CoV-2 R_0_ values were observed in Pakistan (April–June 2020) (54). However, no significant association was detected between ambient UV levels and SARS-CoV-2 R_0_ values in 134 locations in China, USA, and UK (after adjusting for other weather conditions) (55). This observation is supported by findings of another study of people living in 224 Chinese cities, in which no significant associations between ambient UV levels and SARS-CoV-2 R_0_ (January–March 2020) were observed (44). These data may suggest that the transmissibility of SARS-CoV-2 may be less sensitive to UV light than its effects on COVID-19 severity.

An important caveat for many studies described above was that adjustments for possible confounders were not often done. Important confounders to consider that may help resolve these inconsistencies in future studies include consideration of population demographics (e.g., age, gender, GDP, etc.) as well as local environmental and weather factors, particularly temperature and humidity (28, 47, 56, 57). Indeed, a systematic review of 17 studies identified that places with warm and wet climates may reduce spread of SARS-CoV-2 (58). However, these observations are not without controversy, with some studies reporting non-linear influences of temperature and humidity on COVID-19 incidence (59, 60). In one study, lower temperatures but not ambient mean daily UV levels predicted the spread of SARS-CoV-2 in Brazil across Feb–March 2020 (61). The combined effect of environmental conditions is another important consideration, with reports of additional benefits of increased temperatures and UV levels associating with reduced COVID-19 incidence, in a study of 559 grid cells (0.25°) with COVID-19 cases across China (January-February 2020) (62). Other studies report more complex interactions between temperature and ambient UV levels when assessed using data collected from 128 countries and 98 states/provinces (November 2019–April 2020) (28).

### Sun Exposure and COVID-19

Few studies have investigated the impacts of personalized sun exposure (via questionnaire or dosimeter) on COVID-19 incidence or mortality. Asyary and Verswati (63) present some findings from Jakarta (Indonesia) around the impacts of sun exposure on incidence, mortality and recovery from COVID-19, with a significant correlation between recovery and sun exposure reported; however, it is uncertain as to what defined “sunlight exposure” in this study, with these data acquired from the Meterological, Climatological, and Geophysical Agency of Indonesia (63).

### Clinical Trials Testing the Efficacy of UV Light or Sun Exposure on COVID-19

There appear to be few clinical trials underway with the intentions of specifically testing the effects of UV light or sun exposure on COVID-19-related outcomes (ClinicalTrials.gov, searched 18th November 2020 using keywords: ultraviolet/sun exposure/phototherapy (+) COVID-19). One trial identified through this search is assessing the effects of germicidal UVC (254 nm) as a means of decontaminating operating room environments of SARS-CoV-2 (NCT04443803). A second single-armed trial is assessing the capacity of “respiratory application” of UVA to intubated COVID-19 patients to affect bacterial and viral burden (NCT04572399).

### Do Social Distancing Measures Affect Sun Exposure?

In the absence of a vaccine, non-pharmacological interventions such as social distancing measures are being relied on to control spread of COVID-19. These include stay-at-home orders and lockdowns. The strength of these orders and their implementation by governments has varied significantly around world. In a preprint mathematical modeling study, done across 162 countries, every “unit increase” in lockdown severity associated with a 77% decline in COVID-19 growth rates (64). The impacts of lockdown measures on sun exposure levels are largely undescribed, although depending on their severity are likely to be significant. Findings of Moozhipurath and Kraft (2020) suggested that even the “least severe” lockdown orders (i.e., recommendation not to leave home) could mitigate any “protective effects” of the UV Index with COVID-19 growth rates declining by 17% for each unit increase in the UV Index. Of relevance here were substantial reductions in physical activity levels (by ~2 h/week) observed in children and teenagers with obesity living in Verona (Italy) during the lockdown period of March–April 2020 (65). These findings are also suggestive of reductions in sun exposure, as much physical activity for this age group takes place outside. A further concern is that these reductions in physical activity may have adverse effects on overweight and obesity, and potentially increase the risk for a severe COVID-19 event (66). In contrast are findings of increased use of cycling networks in Korea during the social distancing period (March 2020), compared to the same time in 2019, suggestive of more time spent doing physical activity outdoors (67). These differing findings are likely mediated by the nature and strength of lockdowns instituted by specific governments.

Other potential impacts of lockdowns on personalized exposure to UV light could be mediated by changes in air pollution levels, with increases in solar radiance observed in some Indian cities in which particulate matter levels reduced by >50% during lockdowns of March–April 2020 (68). Insolation (the amount of solar radiation reaching the earth's surface) increased by 8% in Delhi (North India) in late March 2020 (during national lockdown) compared to previous years, which was determined by measuring the amount of UV radiation received by solar panels (69). Similarly, in Kannar (South India), solar radiation levels (measured using a pyranometer) during lockdown increased by 7% (68). Anecdotal evidence from media reports suggests that Indian citizens were recommended to seek sun exposure during social isolation in April, 2020 (70). Related to this may be significant correlations observed between *Google Trends* relative search volumes using the words “sunbathing” or “vitamin D” with confirmed COVID-19 cases during the March-April lockdown period (r≤0.668, *p* < 0.001) (71). The combined effects of reduced air pollution and any recommendations to increase personal sun exposure on COVID-19 events are yet to be determined.

## Could UV Light Have Harmful or Beneficial Effects on COVID-19?

### UV Light as a Disinfectant

UV light may have direct effects on the viability of SARS-CoV-2 virus in airborne droplets and fomites, thus reducing both infection rates, and also the size of inoculum in those becoming infected, with correspondingly reduced disease severity (72, 73). Germicidal UVC (254 nm) has photoinactivation effects on coronaviruses (74). It is been proposed as a means of inactivating SARS-CoV-2 in food (75), medical air (76), medical equipment [e.g., phototherapy units (77)], hospital waste and wastewater (78), personal protective equipment [e.g., N95 respirators (79, 80)] and mobile phones (81). UVC may effectively decontaminate N95 respirators, but over time could impact their integrity and wear-ability (82). Potential phototoxic effects of germicidal UVC is an important consideration, especially if utilized by inexperienced users in the home (83), with accidental skin damage reported in health care settings following decontamination of N95 respirators (84).

UVC deactivates SARS-CoV-2 directly. Broad spectrum UVC (200–280 nm) substantially reduced SARS-CoV-2 titers on glass surfaces or N95 respirators by >4-log (85). UV radiation (~280 nm, 37.5 mJ/cm^2^) rapidly inactivated >99% of SARS-CoV-2 virions within 10 s when delivered from a light-emitting diode (86). There may also be viral-inactivating effects of direct sunlight, as simulated solar light, representative of the summer solstice at 40°N (1.6 W/m^2^), inactivated 90% of SARS-CoV-2 virus in artificial saliva after 6–8 min of exposure, when dried onto a steel surface, or when aerosolized (87, 88). Similarly, modeling studies suggest that relatively short durations of exposure to sunlight will inactivate 90% of SARS-CoV-2 virions in as little as 11 min in high UV environments such as Bogota (Columbia; 4.6 °N) (89). However, viral inactivation may not be possible in low UV environments, including at levels experienced in many European cities around the winter solstice (89). These findings have been largely reproduced in other modeling studies (90, 91). While germicidal UVC is likely more effective, there is evidence that other wavelengths of light have germicidal properties, including, far-UVC (222 nm), UVB, UVA, visible (400–700 nm) and infrared (92). It is likely that higher doses of these wavelengths will be required for a similar efficacy as germicidal UVC (254 nm), which was more effective than UVA (365 nm) at inactivating SARS-CoV-2 (93).

### Is UV Dose an Important Consideration?

The health harms of excessive sun exposure—especially that causing sunburn, skin cancers and eye disease—are well-described (14, 94). Too much sun exposure has also been linked to reactivation of persisting viruses (95), an example being herpes simplex virus-1, which resides in latency in trigeminal nerves, and reactivates to form cold sores (herpes labialis) on the lip and facial area. Exposure to high doses of UV (4 × minimal erythemal doses/exposure) induced cold sores in adults with a history of sun-induced cold sores (*n* = 20) (96). The mechanisms behind this UV-induced viral reactivation are not completely understood, but could be linked to suppression of the ability of nerve ganglion-resident CD8+ T memory cells to maintain viral latency (97, 98), and damage to nerve endings in skin (95). After exposure to acute, burning doses of UV light, there can be systemic induction of pro-inflammatory mediators, which may be controlled by the induction of anti-inflammatory pathways (13). This process is likely to be dependent on the underlying health status of each affected individual. For example, for many people with psoriasis, treatment with phototherapies that include exposure to (often non-burning doses of) UV light is likely to be beneficial, reducing skin plaque severity by modulating innate and adaptive immune pathways and suppressing skin inflammation. However, at both low and high doses, UV radiation may injure skin and provoke psoriasis in a subset of patients (13). There have also been calls to limit the use of phototherapy units in dermatological clinics during the pandemic because of concerns related to possible transmission risk and the potential immunosuppressive effects of these therapies (99). Interestingly, biologics, other immune-modifying agents used to treat psoriasis by targeting specific pro-inflammatory proteins such as tumor necrosis factor (TNF), may increase risk for COVID-19. In individuals with psoriatic arthritis living in Italy (*n* = 1,193), use of biologics increased risk for COVID-19 infection (OR 3.4 95% CI 2.2–5.7) and hospitalization (OR 3.6 95% CI 1.5–8.6), but not death (OR 0.4 95% CI 0.0–6.6) (100). Phototherapies can also diminish systemic inflammatory cytokine expression, with narrowband UVB radiation (~311 nm) reducing blood levels of c-reactive protein in people with psoriasis (101). Combined with the potential for UV light to compromise viral-specific T cell responses, high doses of UV light could increase susceptibility for COVID-19, although it is likely that the timing between when UV and viral exposures occur will be an important determinant. In the observational study of Cherrie et al. (48) (described above), the greater reductions from COVID-19 deaths were linked to increases in UV exposure from lower baseline levels. This effect tailed off as environmental UV light increased, suggesting that there may be an upper limit to the levels of UV light associated with reduced COVID-19 mortality.

### Skin Color and COVID-19

Black-American and Latino communities in the United States (102) and Black-British and Asian communities in England (103) have been disproportionately affected by COVID-19. While this partly reflects pre-existing health and social disparities (102), the ~2-fold over-representation of black, Asian and minority ethnic (BAME) healthcare workers over their white co-workers (104) suggest that skin color itself may play a part. Skin is the barrier between the external environment and human biological homeostasis and skin color is the evolutionarily driven adaptation to the varied incident environmental UV light encountered as *Homo sapiens* dispersed around the world (105). The Eurocentric bias of dermatology (106) has resulted in the focus of UV-skin research focusing largely on problems due to over-exposure of lightly-pigmented skin. The contrasting systemic health problems related to underexposure of more heavily-pigmented skin have received less attention (8). We have demonstrated reduced falls in blood pressure with UV exposure in African Americans than white Americans (107) and vitamin D synthesis may be reduced following UVB exposure of individuals with more pigmented skin (108). The reduced penetration of UV light through more pigmented skin (109, 110) suggests that any UV-mediated reductions in COVID-19 mortality would be less in darker-skinned individuals than their geographically co-located compatriots with paler skin color.

### Could Sun Exposure Compromise Effective Vaccination?

The possibility that sun exposure may diminish the efficacy of vaccination has been considered; however, there are limited studies which have addressed this issue, particularly in humans (94, 111, 112). UV-induced immunosuppression could diminish memory-based immune responses to potentially compromise the efficacy of vaccines (111, 113), with exposure dose an important consideration. For example, non-burning doses of solar-simulated light (3 × week; 1.3 × standard erythemal dose during “restoration phase” for 4 weeks, then 1 × week for 8 weeks) did not modify levels of anti-hepatitis B surface antigen-specific antibodies (measured at 12 weeks) when the vaccination was delivered at baseline and after 4 weeks (114). Hart and Norval (112) recently reviewed the capacity of vaccines delivered through UV-exposed skin to induce immune responses, concluding that UV-induced reductions in vaccine efficacy might be possible but that more research is needed to determine the longevity of the effects of UV exposure, and the best skin sites for vaccination.

### Benefits in Those With Cardiometabolic Dysfunction?

Throughout the COVID-19 pandemic, there have been calls for lifestyle interventions which include improvements to diet, increased physical activity and sleep, and controlled sun exposure to help limit cardiometabolic dysfunction to reduce risk for severe events (115). Below we describe factors and mechanisms that may increase risk for severe COVID-19 in those with cardiometabolic comorbidities and describe potential means through which exposure to UV light could be beneficial for those at-risk.

#### COVID-19 and Cardiometabolic Comorbidities

Increased severity and risk of death have been reported across multiple chronic diseases characterized by cardiometabolic dysfunction, including cardiovascular disease, diabetes, hypertension and obesity (3–5). These findings are reminiscent of the associations between influenza (or other respiratory tract) infections and increased risk of heart attack (116). For example, findings from a systematic review and meta-analyses suggested that there is increased risk for hospitalization (OR 2.1 95% CI 1.7–2.6, 19 studies) and death (OR 1.5 95% CI 1.2–1.8, 35 studies) due to COVID-19 for individuals with obesity (4). Similarly, increased risk for hypertension (OR 2.4 95% CI 1.5–3.8) and cardiovascular disease (OR 3.4 95% CI 1.9–6.2) were observed in meta-analyses of 7 studies (117). Other related risk factors include: gender (male) (118, 119), age (older) (120) and ethnicity (121). However, women with polycystic ovarian syndrome may be a sub-population at-risk of COVID-19, due to their increased risk for cardiometabolic comorbidities (122). Younger adults with obesity may also be at increased risk compared to those people of a similar age not living with obesity (123). Lockdown events may have more severe impacts on those living with metabolic dysfunction and obesity, with COVID-19 likely exacerbating pre-existing inequity, racial inequality and the stigma of obesity (124).

A range of likely intersecting mechanisms have been hypothesized that might explain the associations between cardiometabolic diseases and COVID-19 severity risk. The low-grade inflammation which characterizes many of these conditions may exacerbate the cytokine storm, which occurs in the second week of infection with SARS-CoV-2 (125, 126). Excessive oxidative stress and impaired immunity (particularly innate cell activity, such as natural killer cells) and pro-inflammatory pathways (including macrophages, T cells, B cells) may increase susceptibility for severe COVID-19 (126, 127). The ACE2 receptor for SARS-CoV-2 is highly expressed by adipose tissue, and other relevant tissues [e.g., heart pericytes, pancreatic beta-cells (128)], with adipose tissue a potential reservoir for viral infection (125, 126). Entry of the virus may be promoted through pre-existing endothelial dysfunction (129). Of interest, is also the observation that SARS-CoV-2 was detected in the hearts of 61% of autopsied COVID-19 patients (*n* = 39) (130). Increased blood levels of markers of cardiometabolic dysfunction, such as creatinine kinase, aspartate aminotransferase and cardiac troponin 1 have been detected in people who died from COVID-19, compared to those who recovered (131). These observations may be linked to complications of cardiovascular disease induced by COVID-19, such as cardiomyocyte necrosis, hypertension and coronary plaque instability (131) Other hypothesized mechanisms that could increase risk of COVID-19 severity in people with cardiometabolic disease include: diminished lung function; pre-existing lung damage (including lipofibrosis in lungs (132); impaired pancreatic beta-cell function (131); poor responsiveness/capacity for mechanical ventilation; impaired fibrinolysis capacity and pulmonary perfusion; dysregulated endocrine function; and, gut dysbiosis (133, 134). Non-medical factors that associate with increased risk include poverty and over-crowded housing conditions that likely reduce the capacity of individuals to undertake social isolation recommendations, and promote viral spread (125).

#### Potential Means by Which UV Light Improves Cardiometabolic Function

We have previously demonstrated that there are cardiovascular and metabolic benefits of UV exposure, which are linked to the photo-mobilization of nitric oxide from skin stores to the circulation (135, 136). All-cause mortality reduced with increased UV exposure (137) and this reduction is particularly related to reduced cardiovascular deaths (138). Environmental UV levels inversely correlated with myocardial infarctions (139) and blood pressure, with this effect being stronger in white than black Americans (107). COVID-19 deaths are notably more prevalent in black and minority ethnic populations, which may be related to the blunting of UV benefits in darker-skinned individuals (107). The same UV-driven mechanism may drive seasonal variation in development of diabetes and metabolic syndrome, and similar protective effects in people with sun-seeking behaviors (140). Our preclinical studies indicate that regular exposure to low doses (2–3 min, non-burning) of UV light inhibits metabolic dysfunction in mice fed a high fat diet, with beneficial effects on liver lipid levels, glucose tolerance, insulin sensitivity and adiposity (135, 141, 142). Glycemic control may be important to avoid long-term hospital stays, intensive care unit requirement and death from COVID-19 (143).

Some of the metabolic benefits of low dose UV light were mediated via the photo-mobilization of nitric oxide from skin (135, 141, 142); however, there is likely a role for other mediators including molecules affecting neuro-endocrine pathways (144), and those that mediate anti-inflammatory effects of UV radiation (142). It is likely that UV light has cardiometabolic benefits in both males and females, with body weight, fat mass and adiposity limited in both male and female mice exposed to low-dose UV light (135, 141, 142, 145). Similarly, long-term treatment to narrowband UVB (*n* = 3,229) reduced the risk of cardiovascular and cerebrovascular events in both men and women with vitiligo (compared to those with few or no sessions: HR 0.64, 95% CI 0.52–0.78 for all events; *n* = 9,687) from Korea, although associations were more significant in females than males (146). There may also be benefits for sun exposure on blood cholesterol levels, with LDL-cholesterol levels reduced in individuals receiving advice to increase their sun exposure in 2 of 4 identified intervention studies (101), and findings from association studies suggest that sun exposure was associated with increases in HDL (high-density lipoproteins)-cholesterol levels (9). An important consideration here is the potential for outdoor activity to be a confounder in these studies. We have also observed synergistic interactions in the combined effects of physical activity and UV light to improve metabolic health, with reduced liver steatosis and beneficial effects on metabolic and immune pathways observed in brown adipose tissue of mice allowed access to running wheels following exposure to low dose UV light (147). Some commentators also suggest outdoor activity may be a means of improving vitamin D status through the combined effects of physical activity and skin production of vitamin D in response to exposure to UV light (148).

#### Weathering the Cytokine Storm? Beneficial Mediators Produced by Exposure to UV Light

One of the concerning features of COVID-19 is the cytokine storm, which can occur in the second week of the illness. Other related events may be rare inflammatory conditions, such as a Kawasaki-like disease described in older children (i.e., multi-inflammatory syndrome in children; MIS-C), which is characterized by abdominal pain, cardiac dysfunction and shock (149). It is possible that the cascade of anti-inflammatory mediators produced in response to exposure to UV light (13), including nitric oxide (as above) and vitamin D, may act in concert to potentially prevent the COVID-19 cytokine storm and induced inflammation. Indeed, exposure to UV light (and such induced mediators) may reduce circulating pro-inflammatory cytokines linked to COVID-19 cytokine storm events, including interleukin-6 (IL-6) and c-reactive protein (101, 127, 142, 150). Importantly, elevated IL-6 (>70 pg/ml) levels as well as other cytokines (after multivariate adjustment for sex, age, ethnicity, comorbidities) were associated with reduced survival (HR 2.47, *p* < 0.0001) of patients hospitalized for COVID-19 in New York (USA) (151). As we describe in further detail below, the UV-induced mediators, vitamin D and nitric oxide, may have direct anti-viral effects (143, 152, 153), promote the function of mitochondria to limit reactive oxygen species formation, and regulate the renin-angiotensin-aldosterone system (RAAS), to potentially limit the pathogenesis of SARS-CoV-2 (154). Indeed, the increased risk of severe COVID-19 in older people, might be linked to a diminished capacity to produce these mediators with age (155). Below, we review new findings and more specific mechanisms through which UV-induced vitamin D and nitric oxide may be beneficial for reducing risk of severe COVID-19 events.

## Vitamin D and COVID-19

Skin exposure to UVB radiation is necessary for vitamin D synthesis from the precursor 7-dehydrocholesterol. Further chemical conversions in the liver and kidney (and within cells across the body) result in the formation of 25-hydroxyvitamin D [25(OH)D; used to define vitamin D status in blood], and 1,25-dihydroxvitamin D [1,25(OH)_2_D]. Classically, the active hormone, 1,25(OH)_2_D, acts at a cellular level via its interactions with the vitamin D receptor (VDR), then forming a heterodimer with the retinoid X receptor binding to vitamin D response elements across the genome (mainly promoter regions) for genomic (or nuclear) regulation [reviewed by (156)]. There are also non-genomic signaling pathways through which 1,25(OH)_2_D regulates gene expression [reviewed by (157)]. Much commentary has been published already on the potential for vitamin D supplementation to modulate COVID-19 severity, with at least 70 reviews, commentary and perspectives available on PubMed [as of 18th November 2020; e.g., (158–160)]. Some researchers suggest vitamin D supplementation is likely to be of benefit for COVID-19, and that evidence acquired to-date fulfills Hill's criteria for causality in a biological system (161). Vitamin D has a wide range of purported antiviral, immunomodulatory and cardiometabolic effects, which may help combat COVID-19. These include: induction of antimicrobials (cathelicidin, ß-defensins, hepcidin); regulation of lung surfactant levels; endothelial cell function; autophagy (target intracellular pathogens); regulation of innate cytokines (e.g., IL-1ß); inhibition of pro-inflammatory cytokine production (e.g., IL-6, TNF); and, regulation of overactive T cell responses (158, 161). Below we provide more details of possible protective mechanisms of action of vitamin D, review new findings around the associations between blood 25(OH)D and COVID-19, and discuss issues around vitamin D supplementation and clinical trials in this space. Readers are also directed toward a review (162) of the many commentaries already published in the vitamin D and COVID-19 space, which also provides comprehensive details of earlier studies published until the 16th June 2020, as well as another comprehensive review of studies published until 27th September 2020 (161).

### Postulated Mechanisms of Action

#### Is Viral Entry Limited by 1,25(OH)_2_D?

Anti-viral effects of the active metabolite, 1,25(OH)_2_D, have been reported and linked to antimicrobial peptide production by bronchial epithelial cells (163, 164). These effects were observed at relatively high doses of 1,25(OH)_2_D (100 nM), compared to circulating levels (in pM range). It may not be possible to achieve these levels with dietary vitamin D supplementation, and perhaps necessary to consider the possible benefits for non-calcemic analogs of 1,25(OH)_2_D (such as calcipotriol). Newer findings suggest that titers of SARS-CoV-2 grown in nasal epithelial cells were significantly reduced by subsequent treatment with relatively high doses of 1,25(OH)_2_D (i.e., 10 μM) (165). Arboleda Alzate et al. (166) observed that a more physiologically relevant dose of 1,25(OH)_2_D (0.1 nM) limited entry of dengue virus into macrophages, by downregulating the mannose receptor (166). Interestingly, calcitriol [1,25(OH)_2_D] at “higher” doses (from 20 nM) increased ACE2 expression in lipopolysaccharide (LPS)-treated microvascular endothelial cells (167). Further work is needed to determine the *in vitro* and *in vivo* anti-viral effects of 1,25(OH)_2_D treatment of SARS-CoV-2 infections across a range of doses.

#### Immune and Other Pathways That Could Be Regulated by Vitamin D to Reduce COVID-19 Severity

Other researchers have recently reviewed various others means through which vitamin D could limit the impacts of SARS-CoV-2 infection. These include its capacity to regulate autophagy and apoptosis, reduce cytokine hyperproduction and limit lung injury induced by viral infections (168–170). For people infected with human immunodeficiency virus, vitamin D may act as an adjuvant during retroviral therapy with some VDR alleles increasing susceptibility for infection with the human immunodeficiency virus (168). Others hypothesize that vitamin D could combine effectively with interferon (IFN) to control SARS-CoV-2 infection, with vitamin D modulating the expression of IFN-stimulated genes during infection with hepatitis C virus or rhinovirus (171). Vitamin D may limit the proliferation and reduce innate inflammatory responses (e.g., by reducing matrix metalloproteinase expression) by bronchial epithelial cells (153), with potential benefits for epithelial barrier integrity (170). Vitamin D may promote the activity of T regulatory cells and reduce the potential for dendritic cells to activate and expand effector/pro-inflammatory T helper (Th) cell subsets including Th1 and Th17 cells (153). Other postulated mechanisms of action include the capacity of vitamin D to regulate lung and gut microbiota, and inhibitory effects on fibrosis and aging (e.g., via Klotho-pathways) (170). Berthelot et al. hypothesized that there may be benefits for 1,25(OH)_2_D for some manifestations of SARS-CoV-2 infection, including MIS-C and thrombic coagulopathy via downregulation of the STING (stimulator of interferon genes) pathway and production of IFNß (172). At this stage, these are plausible pathways through which vitamin D could be beneficial but are yet-to-be demonstrated for COVID-19.

#### Regulatory Mechanisms by Which Vitamin D May Have Cardiometabolic Benefits

Various pre-clinical experiments have been undertaken to determine mechanisms through which vitamin D may exert benefits for cardiometabolic health. We have previously reviewed mechanisms through which vitamin D may be beneficial for metabolic health (140, 173). Here, we review some of the pathways through which dietary vitamin D or treatment with 1,25(OH)_2_D/related metabolites could modulate cardiovascular health, with the reader directed toward more comprehensive reviews for more information (174, 175).

##### Dietary Vitamin D

Dietary vitamin D may improve cardiovascular health by directly affecting cells of the heart, inhibiting inflammation, preventing fat accumulation and regulating cholesterol pathways. Cardiomyocyte proliferation was increased in hearts of vitamin D-deficient rats, which expressed higher levels of c-Myc protein (176), a known driver of cardiomyocyte proliferation (177). In micro-swine with coronary restenosis (abnormal narrowing of the arteries), dietary vitamin D_3_ reduced expression of circulating pro-inflammatory cytokines (TNF, IFNγ) (178). A vitamin D-deficient hypercholesterolemic diet increased NF-κB (Nuclear Factor kappa-light-chain-enhancer of activated B cells) activation in epicardial adipose tissue and promoted formation of atherosclerotic plaques in pigs (179). Increased numbers of M2-type macrophages with endoplasmic reticulum stress, and more fat accumulation were detected in the aortic roots of vitamin D-deficient mice (180). Finally, dietary vitamin D may suppress circulating triglyceride and LDL-cholesterol levels by regulating enzymatic pathways that modified cholesterol synthesis, through activation of the regulatory Insig-2/sterol regulatory element-binding protein 2 pathway (181).

##### Active 1,25(OH)_2_D

The active vitamin D metabolite/hormone, 1,25(OH)_2_D, may benefit heart health through modulating autophagy, the mammalian target of rapamycin (mTOR) and ß-catenin pathways, apoptosis and endothelial repair. Autophagy in myocardium was normalized by 1,25(OH)_2_D treatment, with increased phosphorylation of AMP-activated protein kinase and reduced phosphorylation of mTOR (182). This reduction in mTOR phosphorylation by 1,25(OH)_2_D was also linked with enhanced cardiac autophagy and the inhibition of the ß-catenin/T cell factor/lymphoid enhancer factor/glycogen synthase kinase 3ß/mTOR pathway (183). Both mTOR and ß-catenin are central mediators of cardiac pathophysiology, while glycogen synthase kinase 3ß is a negative regulator of cardiac hypertrophy. 1,25(OH)_2_D may also improve cardiomyocyte energy metabolism by increasing expression of the SIRT1 enzyme (a NAD+-dependent protein deacetylase) and inhibiting expression of PARP (a DNA-damage sensor) (184). Reduced signs of apoptosis (lower Fas, FasL levels) were observed in the hearts of rats treated with 1,25(OH)_2_D, as well as markers of diabetic cardiomyopathy such as circulating lactate dehydrogenase and creatine kinase (185). 1,25(OH)_2_D may also promote endothelial repair with increased protein of the smooth muscle isoform of the myosin light chain detected in the aortas of rats injected with 1,25(OH)_2_D (186).

##### Other Vitamin D Metabolites

Paricalcitol (a hypocalcemic analog of 1,25(OH)_2_D) may have similar beneficial effects as 1,25(OH)_2_D to decrease markers of fibrosis (187), inflammation (188) and oxidative stress in the heart. These effects included reductions in the enzyme activity of NADPH oxidase and superoxide dismutase (189), and cardiac cholesterol levels (190), and increased serum and cardiac adiponectin levels (190). Reduced atherosclerotic lesions were observed in apolipoprotein E^−/−^ mice administered 25(OH)D_3_ (intragastrically) without causing hypercalcemia, while also increasing regulatory T cells and reducing mature dendritic cells in lesions (191). Neutralization of these regulatory T cells with an anti-CD25 monoclonal antibody increased signs of atherosclerosis (191), highlighting the anti-artherosclerotic potential of these cells.

##### Vitamin D and the Renin-Angiotensin-Aldosterone System (RAAS) Pathway

One important pathway regulated by vitamin D is the RAAS. This has potential importance for COVID-19 with the ACE2 viral entry receptor likely playing important regulatory roles during overactivation of RAAS (192). Furthermore, the capacity for calcitriol [1,25(OH)_2_D] to increase ACE2 expression by microvascular endothelial cells (167) may have implications for the SARS-CoV-2 infectivity and regulation of RAAS by COVID-19. As above, 1,25(OH)_2_D enhanced the expression of ACE2 in LPS-treated lung endothelial cells *in vitro* (from 20 nM) and lungs of LPS-treated rats (167), and in rat brains and microgial cells [1 μM 1,25(OH)_2_D] (193). Conversely alfacalcidiol [an analog of 1,25(OH)_2_D] treatment reduced ACE2 mRNA levels in kidneys of rats with kidney injury (194). Renin is the enzyme which initiates the RAAS cascade. Dysregulation of the RAAS pathway is associated with hypertension and many negative effects upon cardiac and metabolic function, including fibrosis, inflammation, heart failure, aging and diabetic injury (195). Important components of the pathway include angiotensinogen (cleaved by renin to form angiotensin I), the renin receptor and ACE (angiotensin converting enzyme, which cleaves angiotensin I into angiotensin II). Increased expression of some of these components, including renin, the renin receptor, ACE and the angiotensin II type 1 receptor was observed in pancreatic islets of vitamin D-deficient mice (196). Serum renin activity was enhanced in vitamin D-deficient adult LDL-receptor^−/−^ mice fed a high fat diet, with these observations reversed by vitamin D supplementation (180). Although hypothesized (197), is unclear as-yet whether these modulating effects of dietary vitamin D on RAAS will be of benefit for reducing the negative impacts of COVID-19.

### Vitamin D Status and COVID-19

Studies reporting associations between vitamin D status [blood levels of 25(OH)D] and infection with SARS-CoV-2, or morbidity/mortality due to COVID-19 include those that: (i) used previously published data on mean “national” vitamin D status for some European countries (Table 1A); (ii) measured 25(OH)D levels upon hospitalization (for COVID-19) or after PCR test for SARS-CoV-2 (Table 1B); or, (iii) measured 25(OH)D prior to diagnosis of SARS-CoV-2 infection (Table 1C).

Table 1Studies reporting the associations between blood 25(OH)D level or vitamin D status and COVID-19 outcomes.

**Table:** 

**References**	**Time frame**	***N* (countries)**	**Main findings**		**Possible limitations**
**A. ASSOCIATION OF MEAN 25(oh)d LEVELS^A^ AND covid-19 MORTALITY OR CASES (PER MILLION PEOPLE) ACROSS SOME eUROPEAN COUNTRIES**					
Ali (198)	8th April 2020 or 20th May 2020	*n =* 20	- Some evidence for inverse correlation of 25(OH)D with mortality (*r =* −0.44, *p =* 0.05) or cases (*r =* −0.44, *p =* 0.05) (8th April 2020) - Significant inverse correlation of 25(OH)D with cases (*r =* −0.48, *p =* 0.03) but not mortality (*r =* −0.36, *p =* 0.12) (20th May 2020)		- Historical 25(OH)D levels not standardized across countries nor any adjustments made for differences in 25(OH)D assays
Ilie et al. (199)	8th April 2020	*n =* 20	- Some evidence for inverse correlation of 25(OH)D with mortality (*r =* −0.44, *p =* 0.05) or cases (*r =* −0.44, *p =* 0.05)		- No adjustments made for differences in testing rates, social distancing strategies, population demographics, or comorbidities
Laird et al. (200)	(dates not stated, published 7th May 2020)	*n =* 12	- Significant inverse correlation of 25(OH)D with mortality (*r*-value not stated, *p =* 0.046)		
Singh et al. (201)	8th April 2020 and 12th May 2020	*n =* 20	- Some evidence for inverse correlation of 25(OH)D with mortality (*r =* −0.44, *p =* 0.05) or cases (*r =* −0.44, *p =* 0.05) (8th April 2020) - Significant inverse correlation of 25(OH)D with cases (*r =* −0.55, *p =* 0.01) but not mortality (*r =* −0.39, *p =* 0.09) (12th May 2020)		
**#**	**References** ***Location***	**Time frame**	**Population**	**Main findings**	**Possible limitations**
**B. BLOOD 25(OH)D (LIKELY) MEASURED UPON HOSPITALIZATION OR AFTER PCR TEST FOR SARS-CoV-2**.					
1	Abrishami et al. (202) *Tehran, Iran*	28th February−19th April 2020	- *n =* 73 hospitalized with COVID-19 and PCR+ for SARS-CoV-2: - *n =* 12 who died - *n =* 61 who were discharged	- Higher 25(OH)D levels associated with less lung involvement (ß = −0.11 (SE = 0.034), *p =* 0.003 in adjusted model - Vitamin D deficiency [25(OH)D <25 ng/mL] associated with increased risk for mortality (HR 4.2, 95% CI 1.1–16.2) after adjusting for age, sex, comorbidities	- Small sample size - Some uncertainty as to when blood for 25(OH)D was obtained (within 3 days of chest CT?)
2	Arvinte et al. (203) *Thornton, Colorado, USA*	May 2020	- *n =* 21 hospitalized and critically ill with COVID-19 - *n =* 11 survivors - *n =* 10 non-survivors	- No difference in 25(OH)D levels reported between survivors [21.3 (11.3)] and non-survivors (22.8 (7.7) ng/mL [mean (SD)]	- Small sample size - Uncertainty as to when blood for 25(OH)D was obtained - Lack of clarity on 25(OH)D assay - No mention of whether diagnosis with COVID-19 included a SARS-CoV-2+ PCR result
3	Baktash et al. (204) *Slough, UK*	1st March−30th April 2020	- *n =* 105 ≥65-year-olds, including: - *n =* 70 COVID-19+ - *n =* 35 COVID-19–	- 25(OH)D lower in COVID-19+ (mean = 27, IQR 20–47 nmol/L), compared to COVID-19– (mean = 52, IQR 31.5–71.5 nmol/L) (*p =* 0.0008) - Increased ventilation and high dependency unit admission rates, and peak D-dimer blood levels for COVID-19+ with 25(OH)D ≤ 30 nmol/L	- Small sample size - 25(OH)D not measured before illness - Lack of clarity on 25(OH)D assay - Diagnosis of SARS-CoV-2 infection based on positive viral RT-PCR swab or evidence for COVID-19 on a chest radiograph or CT
4	Carpagnano et al. (205) *Bari, Italy*	11th March−30th April 2020	*n =* 42 adults with acute respiratory failure due to COVID-19	- Those with 25(OH)D <10 ng/mL had increased mortality risk (50%) compared to those with 25(OH)D ≥ 10 ng/mL (5%) (*p =* 0.019)	- Small sample size - 25(OH)D not measured before illness
5	Cereda et al. (206) *Pavia, Italy*	March–April 2020	*n =* 129 patients hospitalized with COVID-19 (confirmed PCR+ for SARS-CoV-2)	- Positive association between blood 25(OH)D and mortality risk (OR = 1.7 95% CI = 1.1–2.7, *p =* 0.016) after adjusting for age, sex, blood CRP levels, heart disease, and severe pneumonia	- 25(OH)D measured up to 48 h post-hospitalization
6	D'Avolio et al., (207) *Canton of Tessin, Switzerland*	1st March−14th April 2020	- *n =* 107 adults, including: - *n =* 27 SARS-CoV-2+ - *n =* 80 SARS-CoV-2–	- SARS-CoV-2+ people had significantly (*p =* 0.004) lower 25(OH)D (median = 11.1, IQR 8.2–21.0 ng/mL) than SARS-CoV-2- people (median = 24.6, IQR 8.9–30.5 ng/mL)	- Small sample size - 25(OH)D measured within 7 weeks of the PCR test - Not adjusted for gender or prevalence of co-morbidities
7	De Smet et al. (208) *West Flanders, Belgium*	1st March−7th April 2020	- *n =* 186 patients hospitalized with COVID-19 pneumonia - *n =* 2,717 controls (matched to season, of similar age, stratified for sex)	- 25(OH)D lower in those with SARS-CoV-2 (median 18.6 ng/mL, IQR 12.6–25.3) compared to controls (median 21.5 ng/mL, IQR 13.9–30.8, *p =* 0.002) - Progression of COVID-19 severity (CT stage) significantly associated with increased vitamin D deficiency in males (*p =* 0.001) not females	- PREPRINT - 25(OH)D measured after presentation to hospital - Not adjusted for prevalence of co-morbidities, although no differences in prevalence of chronic lung disease, coronary artery disease and diabetes in people with COVID-19 after stratification for vitamin D deficiency [25(OH)D <20 ng/mL]
8	Faul et al. (209) *Blanchardstown, Ireland*	March 2020	*n =* 33 male Caucasian adults admitted for SARS-CoV-2 pneumonia	- 25(OH)D significantly lower (*p =* 0.03) in those who progressed to acute respiratory distress syndrome (*n =* 12; 27 ± 12 nmol/L, mean ± SD) than those who did not (*n =* 21; 41 ± 19 nmol/L)	- Small sample size - 25(OH)D measured upon presentation to hospital - Not adjusted for prevalence of co-morbidities although none had diabetes or cardiovascular disease
9	Hars et al. (210) *Geneva, Switzerland*	March–April 2020	*n =* 160 older inpatients with COVID-19 including *n =* 95 women, and *n =* 65 men	- Vitamin D deficiency [25(OH)D <50 nmol/L] associated with reduced risk of survival in men (HR 2.5 95% CI 1.0–6.0, *p =* 0.044), but not women in a model that adjusted for age, comorbidities, blood CRP and frailty	- 25(OH)D measured during acute disease - Not all individuals diagnosed with COVID-19 via SARS-CoV-2+ PCR result
10	Hernandez et al. (211) *Spain*	- 10th −31st March 2020 for COVID-19 cases - January–March “past year” for community controls	- *n =* 216 patients hospitalized with COVID-19 - *n =* 197 community controls	- Blood 25(OH)D lower in COVID-19 patients (11.9 95% CI 9.6–14.3) ng/mL) than controls (21.2 95% CI 19.7–22.7) ng/mL) after adjusting for age, smoking, comorbidities, BMI and other (*p* <0.0001) - No significant association of vitamin D deficiency [25(OH)D <20 ng/mL] and COVID-19 severity (OR 1.1 95% CI 0.3–4.8) in adjusted model	- 25(OH)D measured on hospital admission - Some individuals with COVID-19 (*n =* 19) supplemented with vitamin D, although excluded from analyses
11	Im et al. (212) *South Korea*	February–June 2020	- *n =* 50 hospitalized with COVID-19 - *n =* 150 controls matched for age and sex	- 25(OH)D significantly lower (*p* <0.001) in those hospitalized with COVID-19 (15.7 ± 7.9 ng/dL, mean ± SD) than controls (25.0 ± 13.2 ng/dL)	- Small sample size - 25(OH)D measured after admission to hospital - Uncertainty about 25(OH)D assay - Not adjusted/matched for co-morbidities
12	Lau et al (213) *New Orleans, USA*	27th March−21st April 2020	*n =* 20 patients with COVID-19	- 25(OH)D not significantly different (*p =* 0.12) comparing levels from those admitted to ICU (*n =* 13, 19.2 ± 10.8 ng/mL, mean ± SD) to those not (*n =* 7, 29.8 ± 13.3 ng/dL) (*p* = 0.012)	- PREPRINT - Small sample size - 25(OH)D measured after admission to hospital - Not adjusted for age, sex, or co-morbidities - Definition of how COVID-19 was diagnosed not described
13	Luo et al. (214) *Wuhan, China*	- 27th February−21st March 2020 for COVID-19 patients - Same period, 2018–2019 for control group	- *n =* 335 patients hospitalized for COVID-19 - *n =* 560 age- and sex-matched controls group	- ln-transformed 25(OH)D levels significantly less for COVID-19 patients (3.32 ± 0.04 nmol/L, mean ± SD) than controls (3.46 ± 0.02, *p =* 0.014) in model adjusted for age, sex, comorbidities, BMI and other - Vitamin D deficiency [25(OH)D <30 nmol/L] associated with increased COVID-19 severity (OR 2.7 95% CI 1.2–6.0, *p =* 0.014) in adjusted model	- 25(OH)D measured on admission
14	Macaya et al. (215) *Madrid, Spain*	5th−31st March 2020	*n =* 80 patients presenting to hospital emergency with COVID-19	- Vitamin D deficiency [25(OH)D <20 ng/mL] did not significantly associate with increased risk of developing severe COVID-19 (OR 3.2 95% CI 0.9–11.4, *p =* 0.07) in model adjusted for age, gender, obesity, and comorbidities	- 25(OH)D measured on admission or in the past 3 months - Some patients (*n =* 44) supplemented with vitamin D
15	Maghbooli et al. (216) *Tehran, Iran*	Until 1st May 2020	*n =* 235 patients with COVID-19	- Vitamin D sufficiency [25(OH)D > 30 ng/ml] associated with reduced relative risk (RR) of severity (RR 1.6, 95% CI 1.0–2.4, *p =* 0.02), unconsciousness (RR 1.1, 95% CI 1.0–1.1, *p =* 0.03), blood hypoxia (RR 1.3, 95% CI 1.1–1.6, *p =* 0.004), c-reactive protein levels (RR 1.7, 95% CI 1.1–2.6, *p =* 0.01) and lymphocyte percentage <20% (RR 1.4, 95% CI 1.0–1.8, *p =* 0.03)	- 25(OH)D measured after admission to hospital - Not adjusted for age, sex, or co-morbidities - Only 31% of individuals diagnosed with COVID-19 included those with a PCR+ result for SARS-CoV-2 RNA - An expression of concern from the PLoS ONE editors has been published for this paper, highlighting these and other possible issues (217)
16	Mendy et al. (218) *Cincinnati, USA*	13th March−31st May 2020	*n =* 689 patients diagnosed with COVID-19	- Vitamin D deficiency (not defined) significantly associated with hospitalization (OR 1.8, 95% CI 1.1–2.9, *p =* 0.03) or severity (OR 2.0, 95% CI 1.1–3.6, *p =* 0.03) or ICU admission (OR 2.6, 95% CI 1.3–5.1, *p =* 0.03) after adjusting for age, gender, race/ethnicity, smoking	- PREPRINT - Uncertainty about when blood for 25(OH)D was obtained - Uncertainty about 25(OH)D assay
17	Merzon et al. (219) *Tel-Aviv, Israel*	1st February−30th April 2020	*n =* 7,807 members of Leumit Health Services with a previous blood test for 25(OH)D	- 25(OH)D significantly lower for COVID-19+ (*n =* 782, mean = 19.0 ng/ml, 95% CI 18.4–19.6) than COVID-19- (*n =* 7,025, mean = 20.6 ng/ml, 95% CI 20.3–20.8) - low 25(OH)D levels significantly associated with increased risk for infection (OR 1.5, 95% CI 1.1–2.0, *p* <0.001) but not hospitalization (OR 2.0, 95% CI 1.0–4.8, *p =* 0.06) after adjusting for age, sex, SES, comorbidities	- Uncertainty about when blood for 25(OH)D was obtained - Uncertainty about 25(OH)D assay - Some unexpected observations of negative associations between COVID-19+ for some comorbidities (e.g., cardiovascular disease)
18	Panagiotou et al (220) *Newcastle upon Tyne, UK*	(not stated) (published online 3rd July 2020)	*n =* 134 patients hospitalized with COVID-19	- No significant difference (*p =* 0.3) between 25(OH)D levels for those in an intensive therapy unit (*n =* 42, 33.5 ± 16.8 nmol/L, mean ± SD) than those not (*n =* 92, 48.1 ± 38.2 nmol/L) - 25(OH)D levels not associated with mortality (OR 0.97, 95% CI 0.42–2.23)	- Uncertainty when blood for 25(OH)D was obtained prior to COVID-19 testing (measured at “baseline”) - Uncertainty about 25(OH)D assay - Some patients (55.8%) supplemented with cholecalciferol (vitamin D_3_) - Definition of how COVID-19 was diagnosed not described
19	Pizzini et al. (221) *Austria*	Recruited from 29th April 2020	*n =* 109 adults PCR+ for SARS-CoV-2	- Blood 25(OH)D levels not significantly (*p =* 0.12) different between people with mild (*n =* 22, 64 ± 31 nmol/L, mean ± SD), moderate (*n =* 34, 54 ± 19 nmol/L), or severe (*n =* 53, 50 ± 24 nmol/L) COVID-19 when measured 8 weeks after disease onset, although a difference was observed when mild and moderate cases were combined (*n =* 56, 58 ± 25 nmol/L, *p* <0.05) compared to severe only	- 25(OH)D measured 8 weeks after disease onset - Uncertainty about 25(OH)D assay - Some patients (*n =* 10) supplemented with vitamin D - Not adjusted for age, sex, or co-morbidities
20	Radujkovic et al. (222) *Heidelberg, Germany*	18th March−18th June 2020	*n =* 185 COVID-19 patients (*n =* 92 outpatients, *n =* 93 hospitalized patients)	- Vitamin D deficiency [25(OH)D <12 ng/mL] at first presentation associated with increased risk for invasive mechanical ventilation or death (hazard ration (HR) 6.1, 95% CI 2.8–13.4, *p* <0.001) or death only (HR 14.7 95% CI 4.2–52.2, *p* <0.001) after adjusting for age, gender and comorbidities	- 25(OH)D measured on first presentation - Some inpatients (*n =* 12) supplemented with vitamin D
21	Yilmaz and Sen (223) *Diyarbakir, Turkey*	March–May 2020	- *n =* 40 children (3 month−18 years) hospitalized with COVID-19 confirmed by SARS-CoV-2+ PCR - *n =* 45 healthy “matched” controls	- Blood 25(OH)D levels significantly less in children with COVID-19 (13.1, 4.2–69.3; mean, range) compared to controls (34.8, 3.8-77.4; *p* <0.001)	- Uncertainty on when blood for 25(OH)D was obtained - Not adjusted for age, sex, or co-morbidities

**Table:** 

**References *Study Location/Cohort***	**Time frame**	**Population**	**Main findings**	**Possible limitations**
**C. BLOOD 25(OH)D MEASURED BEFORE INFECTION**					
Chodick et al. (224) *Israel*	From 1st Jan 2020	- *n =* 14,520, including: - *n =* 1,317 SARS-CoV-2+ - *n =* 13,203 SARS-CoV-2–	- 25(OH)D levels in those with (23.6 ± 8.6 ng/mL, mean ± SD) SARS-CoV-2 infection, similar to those without (24.1 ± 9.1 ng/mL)	- Lack of clarity on 25(OH)D assay
Hastie et al. (225, 226) *UK Biobank (n = 502,624)*	16th March−14th April 2020	*n =* 449 adults with confirmed COVID-19 and 25(OH)D test (2006–2010) of 348,598 eligible participants	- 25(OH)D not significantly associated with confirmed COVID-19 (OR 1.00, 95% CI 0.998–1.01, *p =* 0.208) after adjustment for ethnicity, age, sex, month of assessment, income, BMI, comorbidities	- 25(OH)D measured some time before COVID-19 pandemic
Hastie et al. (227)*UK Biobank (n = 502,624)*	5th March−25th April 2020	*n =* 656 adults with confirmed COVID-19 and 25(OH)D test (2006–2010) with *n =* 203 deaths of 341,484 eligible participants	- Severe infection and mortality (HR 0.98, 95% CI 0.91–1.06, *p =* 0.696) not significantly associated with 25(OH)D after adjustment ethnicity, age, sex, month of assessment, income, BMI, comorbidities	- Possible “over-adjustment” as BMI and ethnicity both may affect 25(OH)D [commentary from (228)]
Kaufman et al (229) *USA*	9th March−19th June 2020	From *n =* 218,372 tested for SARS-CoV-2	- Significant negative relationship between lower rates of SARS-CoV-2 positivity and higher blood 25(OH)D levels (OR 0.984 for every 1 ng/ml increase in 25(OH)D, 95% CI 0.983–0.986, *p* <0.001) following adjustment for latitude, ethnicity, gender and age (with 25(OH)D levels seasonally adjusted)	- No consideration of co-morbidities
Meltzer et al., (230) *Chicago, USA*	3rd March−10th April 2020	*n =* 489 patients tested for COVID-19 who had their 25(OH)D levels tested in the last year (prior to testing positive for SARS-CoV-2)	- Testing positive for COVID-19 (*n =* 71) was significantly associated with increased risk (RR 1.8 95% CI 1.1–2.1, *p =* 0.02) for being vitamin D deficient [25(OH)D <20 ng/ml] in multivariable analysis	- Uncertainty about 25(OH)D assay
Raisi-Estabragh et al., (231) *UK Biobank (n = 497,996)*	16th March−18th May 2020	*n =* 1,326 with positive COVID-19 test and *n =* 3,184 with negative COVID-19 test all with blood 25(OH)D test (2006–2010)	- No significant association (OR 1.00 95% CI 1.00–1.00, *p =* 0.72) between seasonally adjusted 25(OH)D levels and COVID-19 positivity in a model that also considered sex, age, and BAME ethnicity	- 25(OH)D measured some time before COVID-19 pandemic - Observed significant associations for BAME ethnicity OR 1.8, 95% CI 1.4–2.2, *p =* 9.27 ×10^−7^)

*Identified in PubMed as of 18th November 2020*.

*^a^Mean 25(OH)D levels reported for each country, ranging in year of measurement*.

*Significance defined as p <0.05, 25(OHD = 25-hydroxyvitamin D*.

*CI, confidence interval; CRP, c-reactive protein; HR, hazard ratio; OR, odds ratio; RR, relative risk*.

*BAME, black, Asian and minority ethnic; 25(OH)D, 25-hydroxyvitamin D; CI, confidence interval; HR, hazard ratio; OR, odds ratio; RR, relative risk*.

Significant (or some evidence for) inverse correlations between mean blood levels of 25(OH)D for 12–20 European nations, and COVID-19 cases or mortality in early April 2020 were observed (198–201) (Table 1A). For COVID-19 mortality rates, these inverse correlations were no longer significant by mid-May 2020 (198, 201). Possible limitations of these studies include that there was no standardization in 25(OH)D levels extracted from historical reports, nor adjustments made for differences in 25(OH)D assays. Furthermore, no adjustments were made for country-specific differences in COVID-19 testing rates, social distancing strategies, population demographics, comorbidities and/or other factors. Similar findings were also observed for data acquired from 27 states and union territories of India with inverse correlations observed in mid-August SARS-CoV-2 infection and mortality and mean 25(OH)D levels from previously published data (232).

In Table 1B, we report findings from 21 studies in which 25(OH)D levels were measured in individuals hospitalized for COVID-19 or after confirmed positive test for SARS-CoV-2 (via PCR) (Table 1B). Eleven of these studies reported significantly lower 25(OH)D levels for those with COVID-19 or who were positive for SARS-CoV-2 infection compared to control groups (204, 205, 207–209, 211, 212, 214, 218, 219, 223). Many of these studies were limited by their small sample size, a lack of clarity on what assay was used to measure 25(OH)D, and that 25(OH)D levels were not measured before infection was diagnosed. This is an important consideration as acute illnesses, including COVID-19, may affect blood 25(OH)D levels (228). This concept is supported by findings of 10 studies in which vitamin D deficiency or lower 25(OH)D levels in blood were associated with more severe outcomes in people already diagnosed with COVID-19 (202, 204, 205, 208, 209, 214, 216, 218, 221, 222) (see Table 1B). Other limitations for some studies include a lack of adjustment for differences in gender, age and comorbidities, and that COVID-19 diagnoses did not necessarily include a positive PCR result for SARS-CoV-2. Not all studies were small in sample size, with 2 reporting findings for at least 500 individuals with COVID-19 (218, 219). One of these was a preprint study (218). Merzon et al. (219) reported that low 25(OH)D levels associated with increased risk for infection (OR 1.5, 95% CI 1.1–1.9; *p* < 0.001) after controlling for age, sex, socioeconomic status and comorbidities; however, there were some “unexpected” observations of negative associations between COVID-19+ and some comorbidities (e.g., cardiovascular disease) (219).

In Table 1C is a summary of 6 studies, in which 25(OH)D levels were measured prior to confirmed SARS-CoV-2 infection or COVID-19 diagnosis. Several of these included data from the UK Biobank, with consistent findings for no significant association between COVID-19 infection or mortality and blood 25(OH)D, with adjustments made for sex, age, ethnicity, and/or body mass index, comorbidities and income (225–227, 231). However, it is important to note that baseline 25(OH)D levels were made ≥10 years before COVID-19 diagnosis, with the assumption that vitamin D status remains stable over-time [reviewed by (162)]. These studies have been also been critiqued for “over-adjustment” (228). In one notable very large study (*n* = 218,372), a significant negative relationship was observed between lower rates of SARS-CoV-2 positivity and higher blood 25(OH)D levels (OR 0.984 for every 1 ng/ml increase in 25(OH)D, 95% CI 0.983–0.986, *p* < 0.001) following adjustment for latitude, ethnicity, gender and age (229).

Overall, many of the published studies cited above have significant limitations, and more clarity is needed on what are the nature of the associations of SARS-CoV-2 infection and COVID-19 outcomes with blood levels of 25(OH)D that better consider confounding, and prospectively measure 25(OH)D using standardized assays. Some new cohort studies are underway, which may address these issues, such as COVIDENCE UK, which aims to examine the influence of diet and lifestyle on transmission and severity of COVID-19 in 12,000 people (≥16 years of age) (233). Findings from a systematic review and meta-analysis suggest that vitamin D deficiency significantly associated with an increased mortality risk (OR 1.8, 95% CI 1.1–2.6, *p* = 0.045, including data from 5 studies reviewed above) (234). However, potential sources of misinformation may be arising in this field of research with issues raised (235) about the accuracy of data in some preprint studies, and an Expression of Concern raised by journal editors (217) around the validity of data and its interpretation in another study (216). Another source of concern is an early systemic review and meta-analyses of 7 articles (that were published between 9th April and 20th May 2020), which reported that 25(OH)D levels were significantly lower for those with “poor” (*n* = 634) than “good” (*n* = 669) COVID-19 prognosis (236). Meta-analyses reported in this study are likely flawed with the inclusion of: studies previously in preprint, now seemingly withdrawn [e.g., Alipio et al., 2020, (236)]; data from a letter, which does not cite any original findings (237); and, the inclusion of retrospective studies with small sample sizes that did not adjust for gender, age, comorbidities and other important factors [e.g., (209, 213) of Table 1B].

### Could Vitamin D Supplementation Be a Successful Strategy?

Some early findings suggest patients with COVID-19 (*n* = 105) may be less likely to be supplemented with vitamin D (age-adjusted OR = 0.56, 95% CI 0.32–0.99) than unaffected (*n* = 1,381) individuals, although infected people were also more likely to have obesity and chronic obstructive pulmonary disease in this study of individuals with Parkinson's Disease from Lombardy (Italy) (238). There may be benefits for vitamin D supplementation in reducing the risk of acute respiratory tract infections, as observed in a meta-analysis of high-quality randomized clinical trials (25 trials; *n* = 11,131, adjusted OR 0.88, 95% CI 0.81–0.96) (239). Similar findings were observed in a more recent preprint study, in which meta-analyses of 40 randomized clinical trials (*n* = 30,956), with vitamin D supplementation reducing risk of acute respiratory tract infection (OR 0.89, 95% CI 0.81–0.98), although some publication bias was detected (240). Sub-group analyses in this study point toward better outcomes for those supplemented with daily doses in the range of 400–1,000 IU/day (OR 0.70, 95% CI 0.55–0.89) for 12 months duration (240). No effect was observed on some outcomes such as percentage diagnosed with upper or lower respiratory tract infection, use of anti-microbials, hospitalization and emergency department attendance (240). Vitamin D supplementation may also exert cardiovascular and anti-inflammatory benefits, with meta-analyses pointing toward reduced blood total cholesterol, LDL-cholesterol and triglyceride levels (241) or pro-inflammatory cytokine concentrations (e.g., TNF) compared to placebo, although effect sizes were small (241), especially for those not initially vitamin D-deficient (242). Other meta-analyses suggest that vitamin D supplementation may have beneficial effects on adiposity in certain populations, with sub-group analyses of 20 randomized clinical trials (*n* = 3,153 participants) suggesting reduced body mass index and waist circumference in women living in Asian countries supplemented for ≥ 6 months (243). Multi-omic analyses of published datasets have identified vitamin D (among a suite of other candidates) as a potential prophylactic agent for COVID-19 (244). However, being treated with vitamin D, did not significantly change the incidence of COVID-19 in a study of >2,000 people treated for non-inflammatory rheumatic conditions (Mar-May 2020) (245).

### Vitamin D Dose Considerations

One ongoing issue in vitamin D supplementation studies concerns what are effective and safe dosing for optimal health. Significant debate has been published across multiple journals, including the *Irish Medical Journal* (246–248) and *Nutrients* (160, 249, 250) on what are safe doses of vitamin D that could provide protection from COVID-19. Some suggest that very high bolus doses are likely to be safe in ventilated, critically ill patients, based on reports that doses of 250,000–500,000 IU vitamin D were associated with reduced length of stay in hospital, and increased blood oxygen levels [reviewed by (251, 252)]. Others are more circumspect around dosing, calling for multi-center randomized controlled trials to help define safe and effective doses for COVID-19 (253). There may be ill effects of “high” daily doses of vitamin D on markers of cardiovascular health. For example, increased need for mechanical circulatory support implants was observed in adults with heart failure (*n* = 400) supplemented with 4,000 IU vitamin D/day for 3 years (HR 2.0, 95% CI 1.0–3.7) (254). These implants are a last option to prevent death in people with end-stage heart failure and their increased use was linked to vitamin D-induced hypercalcemia (254). Indeed, long-term (≥12 months) treatment with “high” dose vitamin D (≥2,800 IU/day) may increase risk of hypercalcemia (255). Further controversy lies in the frequency of dosing (e.g., daily vs. bolus monthly), although for acute respiratory infections, meta-analyses point toward increased efficacy of daily dosing with 400–1,000 IU vitamin D (240). Clinical trials that compare the efficacy of vitamin D supplementation to limit COVID-19 severity and measure safety outcomes across range of daily doses are needed.

### Factors Modulating the Potential Protective Effects of Vitamin D and COVID-19

Gender is an important consideration, with increased risk of severe COVID-19 linked to being male (155). The reasons for this observation are not fully understood, but could include sex-linked differences in immunity, meta-inflammation responses, expression of ACE2 and risk of developing acute respiratory distress syndrome (ARDS) (155, 256–258). There may be gender differences in the capacity of vitamin D to regulate immune responses, including increased capacity to induce T regulatory cells and immunosuppressive cytokines such as interleukin-10 in females [reviewed in (259)]. These responses may be mediated via sex hormones such as estrogen and progesterone. Indeed, vitamin D may cooperate with progesterone to regulate immunity (potentially by upregulating the VDR) to control pro-inflammatory cytokine expression (260). Of importance here were recent findings that men (but not women) with vitamin D deficiency [25(OH)D < 50 nmol/L] were at increased “risk” of dying due to COVID-19 (210), with similar findings in a second study (208). Age may be another important factor to consider as a significant interaction between age and vitamin D deficiency was observed for associations with COVID-19 severity in one study (215). Other important considerations include genetic influences, with significant associations between polymorphisms in the vitamin D-binding protein and the prevalence and mortality due to COVID-19 reported (261). How ethnicity affects our capacity to make vitamin D after exposure to sun exposure (or UVB radiation) is a further important factor, which has been discussed (121, 262). Some researchers hypothesize there may be important links between genetic deficiencies in glutathione synthesis (via less G6PD enzyme activity) and vitamin D deficiency in African American people, compared to others, and protection from oxidative stress induced by COVID-19 (263). Further data are needed on the influence that skin color and type (e.g., Fitzpatrick) have on COVID-19-related outcomes.

### Clinical Trials Testing the Efficacy of Vitamin D Supplementation

Early findings from small clinical trials and experimental studies are suggestive of some benefit for vitamin D supplementation for lessening COVID-19 severity. When the effects of standard care with and without oral calcifediol [25(OH)D; 0.532 mg on day of admission, 0.266 mg on days 3 and 7 then weekly] were compared in 76 people admitted to a Spanish hospital for COVID-19, reduced risk for admission to intensive care was observed for those treated with calcifediol (OR 0.03, 95% CI 0.003–0.25, after adjusting for prevalence of hypertension and type-2 diabetes) (264). From two “quasi-experimental” French studies, regular supplementation with “bolus” vitamin D [e.g., 80,000 IU vitamin D every 2–3 months (265)] prior to disease onset, reduced the risk of death from COVID-19 in both hospitalized patients [*n* = 77: HR 0.07 95% CI 0.01–0.61 (266)], and people living in a nursing home [*n* = 66: HR 0.11 95% CI 0.03–0.48 (265)] with models adjusted for age, gender and other factors. A limitation of these studies was that not all SARS-CoV-2 infections were confirmed by PCR. The effects of daily dosing with 60,000 IU cholecalciferol (vitamin D_3_) daily for at least 7 days were compared to placebo for 40 asymptomatic or mildly symptomatic adults who were SARS-CoV-2+ (by PCR), vitamin D-deficient and did not require later ventilation nor had a “significant” comorbidity (267). More individuals treated with cholecalciferol (*n* = 16) were negative (62%) for SARS-CoV-2 RNA by PCR than for those receiving placebo (*n* = 24) (21%, *p* < 0.018) at the 14 day time-point (267).

Other trials are underway testing the capacity of vitamin D supplementation (alone) to reduce the severity of COVID-19, with at least 25 registered on ClinicalTrials.gov as of the 18th November 2020. These are mainly studies recruiting adults, testing the effects of oral vitamin D [or 25(OH)D] supplementation on a range of outcomes, including: SARS-CoV-2 infection rates (including asymptomatic), COVID-19 hospitalization rates and length of stay, admission to intensive care units, requirement for oxygen/ventilation support, symptom severity, cardiovascular events (e.g., heart attack, stroke), sepsis, inflammatory markers, 25(OH)D levels and anti-SARS-CoV-2 antibody titres in blood, and adverse events. Supplementation doses and regimens also vary, ranging from “lower” daily doses of 1,000 IU vitamin D/day (e.g., NCT04476680) to bolus vitamin D administration (e.g., 20,000 IU/day for 3 days and then 6,000 IU day for 12 months; NCT04482673). Other related approaches include supplementation via combined nutritional support, including one very large clinical trial planning to recruit 80,000 individuals in Norway to test the preventative effects of cod liver oil (via 5 mL doses containing 10 μg vitamin D_3_) (NCT04609423).

## Nitric Oxide and COVID-19

Nitric oxide levels are locally and systemically regulated by multiple pathways. Nitric oxide is produced via the activity of nitric oxide synthases [NOS; e.g., induced (iNOS); endothelial (e)NOS], which convert oxygen and L-arginine into nitric oxide and L-citrulline. There are also dietary sources of inorganic nitric oxide precursors, nitrite (${\text{NO}}_{2}^{-}$) and nitrate (${\text{NO}}_{3}^{-}$), which occurring naturally in green leafy vegetables, beetroot and seaweed (268). Nitrate is reduced to nitrite through the activity of tongue microflora and enters the circulation via saliva and for further reduction to nitric oxide in various tissues, including the lung (269). Impaired production of local nitric oxide, through endothelial dysfunction, and reduced expression of eNOS, and decreased bioavailability of nitric oxide occurs in older men and people with comorbidities such as obesity and hypertension, and is hypothesized to increase mortality due to COVID-19 (270). Genetic differences in the expression of eNOS may underpin ethnic differences in susceptibility for severe COVID-19 (271). However, nitric oxide can have more direct anti-viral effects, including inhibition of the replication of a number of viruses at an early stage and activating innate immune pathways for more generalized anti-viral functions (152). Exposure to UV radiation is also another means of increasing the bioavailability of nitric oxide through photo-release from stores in the skin (272). Nitric oxide “bioactivity” is then mobilized to the systemic circulation (as nitrite) to potentially promote vasodilation and reductions in blood pressure (136). Below we describe in more detail potential mechanisms of action of nitric oxide that could reduce severity of COVID-19, including anti-viral effects, as well as anti-inflammatory and cardiometabolic benefits. We then discuss other ways of improving nitric oxide bioavailability that are being considered for prevention or treatment of COVID-19, including provision of inhaled nitric oxide or via dietary supplementation (with nitrate/nitrite) and other methods.

### Postulated Mechanisms of Action

#### Anti-viral Effects

Nitric oxide has general anti-viral actions as well as specific inhibitory effects on coronaviruses. *In vitro* replication of SARS-CoV-2 was inhibited by treatment of Vero-E6 cells with 200–400 μM of the nitric oxide donor SNAP (S-nitroso-N-acetylpenicillamine) (273). This effect might be related to the capacity of nitric oxide to promote S-nitrosylation of cysteine groups (274), a process which inhibits the action of viral proteases (275). Indeed, the protease activity of the SARS-CoV-2 3CL cysteine protease was inhibited by ≥100 μM SNAP (273). Nitric oxide also inhibits replication of SARS-CoV-1 (276) and does this by two S-nitrosylation-dependent pathways. Firstly, S-nitrosylation of the SARS-CoV-1 spike protein prevents the post-translational palmitoylation needed for it to fuse with its receptor (i.e., ACE2) (277). Secondly, early viral replication is blocked by actions on the SARS-CoV-1 cysteine proteases. The spike protein of SARS-CoV-1 is highly homologous to that of SARS-CoV-2 (278, 279), suggesting that nitric oxide will similarly limit binding to ACE2 by SARS-CoV-2. Serine proteases including TMPRSS2 need to prime the spike protein of SARS-CoV-2 to allow cell entry (280), a process particularly sensitive to nitrosylation. The nitric oxide donor, furoxan, is hypothesized to act as a protease inhibitor of SARS-CoV-2 (281).

#### Anti-inflammatory Effects

SARS-CoV-2 is responsible for a wide range of extra-pulmonary manifestations. These may reflect direct viral tissue damage, and include widespread endothelial cell damage, thrombo-inflammation and immune dysregulation (282). Nitric oxide has multiple functions and at low levels such as those produced by the constitutive nitric oxide synthases, it is important for healthy endothelial function (283) and is anti-thrombotic (284). One important immune cell regulated by nitric oxide are macrophages, which are “repolarized” from type-1 (M1) toward a type-2 phenotype (M2), effectively neutralizing pro-inflammatory mediator and reactive species production by these cells [reviewed by (285)]. Nitric oxide mobilized from the skin to the systemic circulation following exposure to UV radiation shows the same bioactivity as these constitutive levels of nitric oxide release and would be expected to have analogous anti-inflammatory actions (136). The human transcriptome shows strong seasonality, with almost one third of all genes involved, with broad up-regulation of anti-inflammatory genes observed in summer in healthy volunteers, a process which may be regulated by sunlight exposure (286).

#### Mechanisms of Cardiometabolic Protection by Nitric Oxide

While physiologically relevant levels of nitric oxide have protective effects, nitric oxide in excess is damaging (287). For example, high concentrations of nitric oxide cause nitrosative stress and mitochondrial injury, while too little can impair mitochondrial biogenesis and function (288). Below we review mechanisms (identified in pre-clinical studies) by which “direct” dietary sources of nitric oxide (i.e., inorganic nitrate and nitrite, or, deficiency) may modulate cardiovascular health. For review of the effects of nitric oxide on metabolic outcomes, please see other reviews (140, 173, 289).

##### Dietary Nitrate

Dietary nitrate may have anti-inflammatory, -fibrotic and -oxidative benefits to improve heart health. Long-term deficiency in nitrate (or nitrite) through dietary restriction caused hypertension, endothelial dysfunction, metabolic syndrome and gut dysbiosis (reduced bacterial diversity, more Actinobacteria) (290). These effects were all reversed by nitrate supplementation. Dietary supplementation with sodium nitrate reduced blood pressure in rats fed a high carbohydrate, high fat diet and was accompanied by reduced inflammation and fibrosis in the left ventricle, with modified cardiac expression of related mRNAs (i.e., reduced *Ctgf*, *Mcp1*α, and *Mmp2*; increased *Ppar*α) (291). Sodium nitrate also inhibited the formation of superoxides in heart tissue and mesenteric arteries of rats with age-related hypertension (292).

##### Dietary Nitrite

Similar to dietary nitrate, nitrite may also have protective effects via pathways that reduce fibrosis and oxidative stress and impair remodeling of cardiovascular tissues. Dietary nitrite reduced the extent of fibrosis and both vascular (187, 293) and cardiac (294, 295) remodeling. The hypotensive and cardiobeneficial effects of dietary nitrite associated with activation of the SIRT3-AMPK pathway in skeletal muscle (187). SIRT3 is a mitochondrial regulator of reactive oxygen species. Nitrite suppressed respiration and reactive oxygen species production in mitochondria and improved their efficiency as an ongoing source of ATP during ischemic attack (289). Dietary nitrite may limit ventricle hypertrophy by suppressing phosphorylated-Akt (a mediator of cardiomyocyte death) levels in the hearts of mice (294).

##### Dietary Nitrite/Nitrate and the Renin-Angiotensin-Aldosterone System (RAAS) Pathway

Like vitamin D, dietary nitrite and nitrate may regulate the RAAS pathway to promote heart health. Inorganic nitrite prevented cardiac remodeling and modulated elements of the RAAS, including; plasma levels of angiotensin II, and cardiac mRNA levels of the angiotensin II type 1 receptor (295). Similarly, sodium nitrate “normalized” angiotensin II signaling in arteries of rats with age-related hypertension (292).

### Clinical Trials Assessing Nitric Oxide

#### Inhaled Nitric Oxide

Inhaled nitric oxide has been proposed for treatment of COVID-19 in several commentaries and reviews (296–299) for its potent selective pulmonary vasodilation and bronchodilation effects, safety profile, and extensive use in treating respiratory disease. Inhaled nitric oxide was previously tested in a very small trial of people with SARS (*n* = 14, China), and significantly reduced the “spread of lung infiltrates” measured via chest radiography (300). Systematic reviews suggest no consistent benefits for inhaled nitric oxide in reducing mortality due to ARDS, although it may improve blood oxygenation and limit pulmonary hypertension [reviewed by (285, 301)]. Even so, inhaled nitric oxide is already recommended and used by some clinicians for treatment of children infected with SARS-CoV-2 (302, 303), and is likely already being combined with other therapies, such as treatment of patients in the “supine and prone” position (i.e., on the tummy) (302, 304), which its self may promote nitric oxide levels in dorsal lung tissue (305).

Possible benefits of inhaled nitric oxide therapy have been reported in case studies of people with COVID-19, including “home therapy” (296, 306), and via portable systems developed for potential use in out-patient settings [reviewed by (285)]. In small clinical studies, inhaled nitric oxide (at doses of 10–25 ppm) did not significantly modify the PaO_2_/FiO_2_ ratio (a measure of oxygenation) in ventilated patients with COVID-19, who had refractory hypoxemia (307–309). At a slightly higher dose, inhaled nitric oxide (starting at 30 ppm) improved oxygenation of 21 of 39 non-intubated individuals with COVID-19 (310). A number of clinical trials are underway (*n* = 20, ClinicalTrials.gov, 18th November 2020 using keywords: nitric oxide (+) COVID-19), examining potential benefits of inhaled nitric oxide in both mechanically-ventilated (311) and spontaneously-breathing (312) patients at “higher” (140–180 ppm) (312) or “lower” doses (e.g., 20 ppm, NCT04388683). A range of outcomes are being tested, including: oxygenation (311); disease progression requiring intubation (312); hospitalization rate and length of stay; mortality; respiratory failure; recovery; blood inflammatory markers; SARS-CoV-2 burden; bacterial load in sputum; adverse events (e.g., kidney failure); lung spirometry; and, quality of life. Other clinical trials underway include those administering a “nitric oxide releasing solution/drug” intranasally (e.g., NCT04443868, NCT04337918), or testing inhalation of a “nitric oxide generating solution” (RESP301) (e.g., NCT04460183) to prevent or treat COVID-19. One trial is testing the capacity of nitric oxide (30 mg)-generating lozenges taken twice daily on blood pressure and dizziness in participants with a recent COVID-19 diagnosis (NCT04601077).

#### Dietary Supplementation and Other Approaches

Despite possible benefits for cardiovascular health, nitrite has complex dose-dependent effects on vasodilation and systemic blood pressure and toxicity issues, which may prevent it from being administered chronically in humans (313, 314). Indeed, no active clinical trials were identified in which dietary nitrite or nitrate on COVID-19 outcomes are being assessed (searching ClinicalTrials.gov, 18th November 2020, using keywords: nitrite (OR) nitrate (+) COVID-19). Other concerns include observations that nitrate can attenuate vascular responses to nitrite, reducing the capacity of nitrite to lower blood pressure (as observed in Wistar rats), by inhibiting xanthine oxidoreductase, the enzyme that catalyzes the formation of nitric oxide from nitrite (and nitrate) (315). In addition, many of the studies detailed above were performed in male experimental animals, and as the cardioprotective effects of estrogen (and other endogenous mediators) may occur through nitric oxide-specific pathways (316, 317), it will be important in the future to consider how translatable the preclinical findings (from mainly male animals) are for both men and women. Other means of increasing bioavailability of nitric oxide for treatment of people with COVID-19 are being considered, including the application of nitric oxide donors (e.g., SNAP, RRx-001), novel technologies such as nanoparticles that release nitric oxide (285, 318), and via the diet (e.g., promoting beetroot intake) (319).

## Future Directions and Concluding Comments

It is clear that there is considerable interest in the potential for UV light, and induced mediators such as vitamin D and nitric oxide to modulate the incidence and severity of COVID-19. Data collected to-date suggest that ambient levels of both UVA and UVB may be beneficial for reducing incidence or severity of COVID-19, although more studies are needed that better consider possible environmental and population-based confounding. Currently unresolved are the nature of the associations between blood 25(OH)D and COVID-19, with more prospective data needed that better consider lifestyle factors, such as physical activity and personal sun exposure levels, and other confounders (e.g., comorbidities). There has been little data collected to-date that considers personal sun exposure and COVID-19-related outcomes. This is an important consideration as ambient UV levels do not always strongly correlate with an individual's sun exposure [e.g., when determined via dosimeter; *r* = 0.2 (320)]. Clinical trials testing the efficacy of new vaccines, and/or vitamin D supplementation, could provide additional opportunities to collect information on personal sun exposure levels, and its association with COVID-19 outcomes, as well as interactions of sun exposure with the impacts of vaccination and vitamin D supplementation. Measuring sun exposure may be important for the development of informed and evidence-based advice, particularly for people at-risk of severe COVID-19 events. New trials will likely consider the combined effects of vitamin D supplementation or inhaled nitric oxide with other successful treatments [e.g., corticosteroids such as dexamethasone (321)]. We highlight that UV light, and induced mediators, may exert anti-SARS-CoV-2 effects, with protective effects hypothesized for respiratory and cardiometabolic health during COVID-19; however, more evidence is needed.

## Author Contributions

SG and RW conceived, designed, and wrote this review together. Both authors contributed toward the critical analyses of manuscripts discussed and have given their final approval for this version of the paper to be published.

## Conflict of Interest

RW is a member of the Scientific Advisory Board of AOBiome LLC, a company commercializing ammonia-oxidizing bacteria for use in inflammatory skin disease. The remaining author declares that the research was conducted in the absence of any commercial or financial relationships that could be construed as a potential conflict of interest.

## Abbreviations

1,25(OH)_2_D: 1,25-dihydroxyvitamin D
25(OH)D: 25-hydroxyvitamin D
ACE2: angiotensin-converting enzyme 2
ARDS: acute respiratory distress syndrome
COVID-19: coronavirus disease of 2019
GDP: gross domestic product
IFN: interferon
IL-6: interleukin-6
LDL-: low-density lipoproteins
LPS: lipopolysaccharide
MIS-C: multi-inflammatory syndrome in children
mTOR: mammalian target of rapamycin
NOS: nitric oxide synthase
RAAS: renin-angiotensin-aldosterone system
SARS-CoV-2: severe acute respiratory disease coronavirus-2
Th: T helper cell
TNF: tumor necrosis factor
VDR: vitamin D receptor
UV: ultraviolet.
